# Reappraisal of the Variations in the Origin of the Obturator Artery With Clinical and Developmental Perspectives: A Cadaveric Analysis

**DOI:** 10.7759/cureus.68728

**Published:** 2024-09-05

**Authors:** Apurba Patra, Navita Aggarwal, Priti Chaudhary, Vandana Tiwari

**Affiliations:** 1 Anatomy, All India Institute of Medical Sciences, Bathinda, Bathinda, IND

**Keywords:** anatomical variations, obturator artery, pelvic cavity, pelvic vasculature, surgical procedures

## Abstract

Background

The obturator artery (OA), typically originating from the anterior division of the internal iliac artery (ADIIA), shows significant variability in its origin. Such variations can present clinical challenges during pelvic surgeries, potentially causing unnoticed bleeding and complicating effective treatment. This study aims to thoroughly document the diverse anatomical variations of the OA and explore their implications for surgical practice.

Materials and methods

Forty-eight hemipelvis specimens from adult human cadavers were dissected. The origin of each OA was meticulously documented, photographed, and analyzed descriptively.

Results

In 38 specimens (79.2%), the OA originated from the IIA. It branched off at various levels from either the ADIIA or the posterior division of the IIA (PDIIA), either individually or in combination with other named branches. In nine cases (18.8%), the OA originated directly from the external iliac artery (EIA), either as a distinct branch or alongside the inferior epigastric artery (IEA). Additionally, one specimen (2%) exhibited a dual origin involving both the ADIIA and the IEA.

Conclusion

These findings highlight the frequent anatomical variations in the origin and pathway of the OA. Understanding these variations is crucial for accurately assessing pelvic anatomical relationships, which is essential for effective surgical planning and ensuring procedural safety. This knowledge is particularly important during vascular and surgical procedures, as it can impact the risk of bleeding and the effectiveness of treatment strategies.

## Introduction

The abdominal aorta terminates by dividing into the left and right common iliac arteries (CIA). These further branch into the corresponding external iliac artery (EIA) and internal iliac artery (IIA) on each side. The EIA eventually becomes the femoral artery (FA) in the thigh and supplies blood to the lower limb. The IIA divides into anterior and posterior divisions and supplies blood to the structures in the region of the pelvis, gluteal, and perineum [1]. The obturator artery (OA), usually arising from the anterior division of the IIA (ADIIA), traverses the obturator foramen (OF) alongside the obturator nerve (ON) and supplies the ilium bone through a nutrient branch, as well as the iliacus, obturator externus, and muscle of the adductor compartment of the thigh [2].

Variations in the origin of the OA are well-documented in human anatomy studies [1-8]. These variations, known as aberrant obturator arteries (AOAs), often involve atypical origins and locations such as the IEA or EIA. A variant, termed “corona mortis” or “crown of death,” presents a plausible hazard during surgical procedures or pelvic fractures, where a vascular anastomosis between the IEA and the OA may course over the superior pubic ramus, increasing the risk of accidental injury during ligation [4,5].

Studies have highlighted the importance of recognizing and understanding these variations, as unfamiliarity with AOAs can lead to a layer of complications during clinical interventions such as gynecological procedures, urological interventions, and endoscopic hernioplasty in the pelvic region [9-11]. The cases of trauma involving pelvic injuries require rapid and accurate assessment of the vascular anatomy of the area to determine the extent of damage and to guide appropriate interventions. Also, familiarity with AOA is equally vital for properly disseminating knowledge to anatomical instructors. Advanced imaging techniques, such as magnetic resonance angiography, conventional angiography, or CT angiography, are employed to visualize and map the vascular anatomy before undertaking these procedures [12]. These imaging techniques allow precise planning and safer execution of embolization and revascularization interventions in the pelvis [13,14]. Despite existing literature, there remains a need for ongoing research and documentation of such anatomical variations to foster a comprehensive understanding of variations in abdominal and pelvic vascular anatomy, particularly among diverse demographic groups in the Indian population. This will allow healthcare professionals to provide the best possible care to their patients. With such a background, the present study investigated the variabilities in the origin of OA among the study population and their potential implications in pelvic surgery, thus helping improve clinical outcomes in Indian patients undergoing procedures in the relevant anatomical region.

## Materials and methods

The present institution-based observational study was done on 48 hemipelvis specimens (30 males and 18 females) obtained from 24 formalin embalmed adult human cadavers (15 males and nine females) of north India aged between 40 and 78 years. These cadavers were sourced from the departmental voluntary body donation program for teaching and research purposes. Hence, the study protocol was exempted from institutional review board approval. The specimens without any history of pelvic trauma, surgery, or any visible deformity were included in the study. Cadavers with a history of previous pelvis surgery, gross obesity, and autopsied bodies were excluded from the study. After applying the inclusion and exclusion criteria, these hemipelvis were dissected to look into the origin of the OA and its variations.

Dissection of the pelvic region was done per the steps given in Cunnigham’s manual for practical dissection of the abdomen and pelvis [15]. A systematic approach was employed, progressing from the superficial pelvic structures to the deep. Pelvic viscera in the right and left iliac fossa were carefully reflected to access the posterior abdominal wall. Dissection continued to expose the CIA. The point of division between the EIA and IIA was identified, and the branches of IIA were traced. The OA was identified. The origin, course, and relationships with adjacent structures were meticulously recorded separately as part of the study data by two authors, and photographs were captured using an iPhone 11 Pro camera. The length of the OA (distance between the point of origin and the entry point into the obturator foramina) was measured with the help of digital vernier calipers (Mitutoyo Japan).

The collected morphological and morphometric data was entered into the Microsoft Excel sheet and analyzed statistically. Descriptive statistics were used to summarize the frequency and percentage of OA variations.

## Results

Table 1 summarizes the source of OA origin in male and female cadavers across the right and left pelvic halves.

**Table 1:** Comparisons of various sources of origin of OA in the cadaveric pelvic halves observed in the present study EIA, external iliac artery; IIA, internal iliac artery; OA, obturator artery

Origin of OA	Male, N (%)		Female, N (%)		Total, N (%)
	Right, 15 (100%)	Left, 15 (100%)	Right, 9 (100%)	Left, 9 (100%)	
IIA	11 (73.3%)	12 (80%)	7 (77.8%)	8 (88.9%)	38 (79.2%)
EIA	3 (20%)	3 (20%)	2 (22.2%)	1 (11.1%)	9 (18.8%)
Dual origin (from both IIA and EIA)	1 (6.7%)	0 (0%)	0 (0%)	0 (0%)	1 (2%)

**Figure 1:**
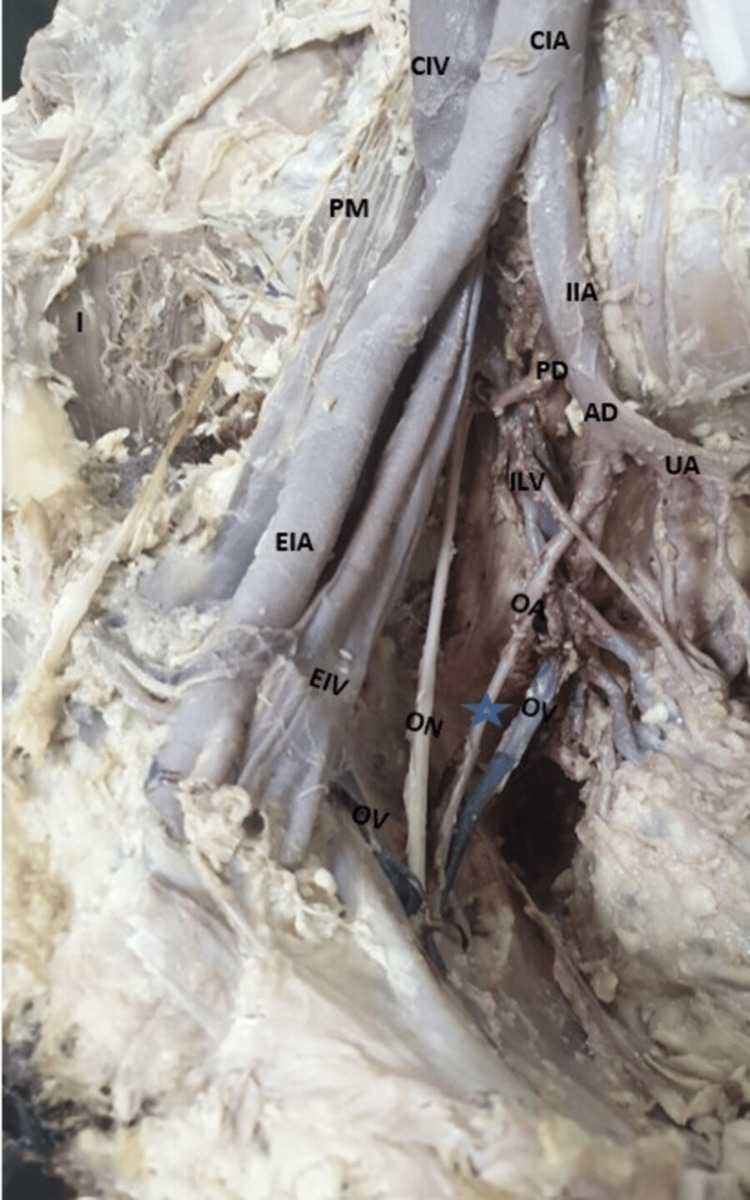
Right side of the pelvis showing the origin of the OA from the AD of the IIA AD, anterior division; CIA, common iliac artery; CIV, common iliac vein; EIA, external iliac artery; EIV, external iliac vein; I, iliacus; IIA, internal iliac artery; IIV, internal iliac vein; OA, obturator artery; ON, obturator nerve; OV, obturator vein; PD, posterior division; PM, psoas major muscle; UA, umbilical artery

**Figure 2:**
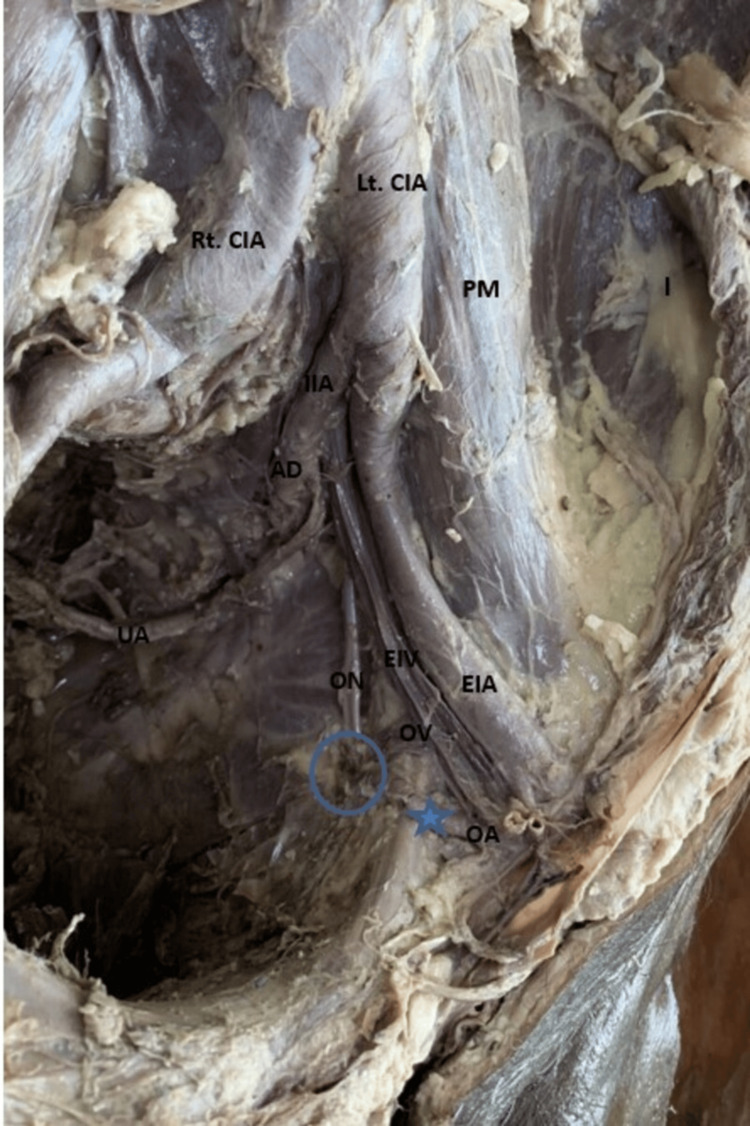
Left side of the pelvis showing the origin of the OA from the EIA AD, anterior division; CIA, common iliac artery; CIV, common iliac vein; EIA, external iliac artery; EIV, external iliac vein; I, iliacus; IIA, internal iliac artery; IIV, internal iliac vein; OA, obturator artery; ON, obturator nerve, OV, obturator vein; PD, posterior division; PM, psoas major muscle; UA, umbilical artery

**Figure 3:**
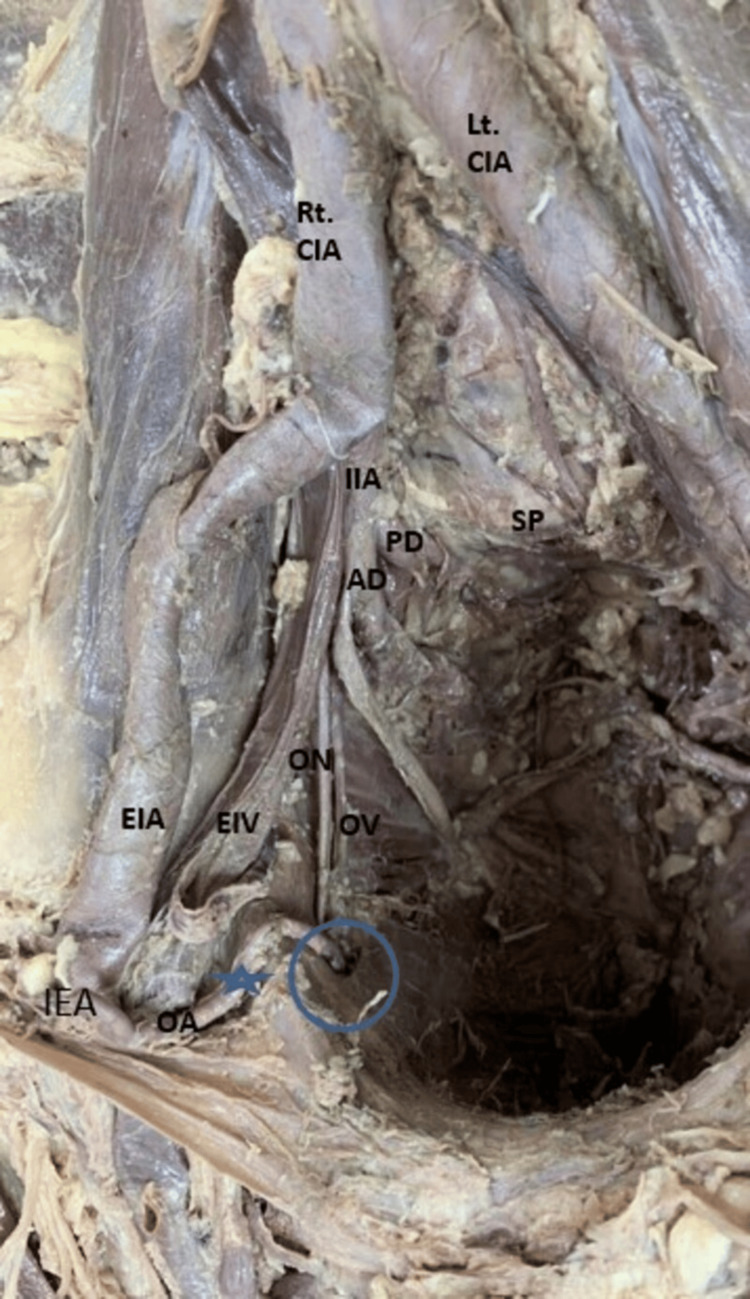
Right side of the pelvis showing the origin of the OA from the IEA AD, anterior division; CIA, common iliac artery; CIV, common iliac vein; EIV, external iliac vein; I, iliacus; IEA, inferior epigastric artery; IIV, internal iliac vein; OA, obturator artery; ON, obturator nerve; OV, obturator vein; PD, posterior division; PM, psoas major muscle; SP, sacral promontory; UA, umbilical artery

**Figure 4:**
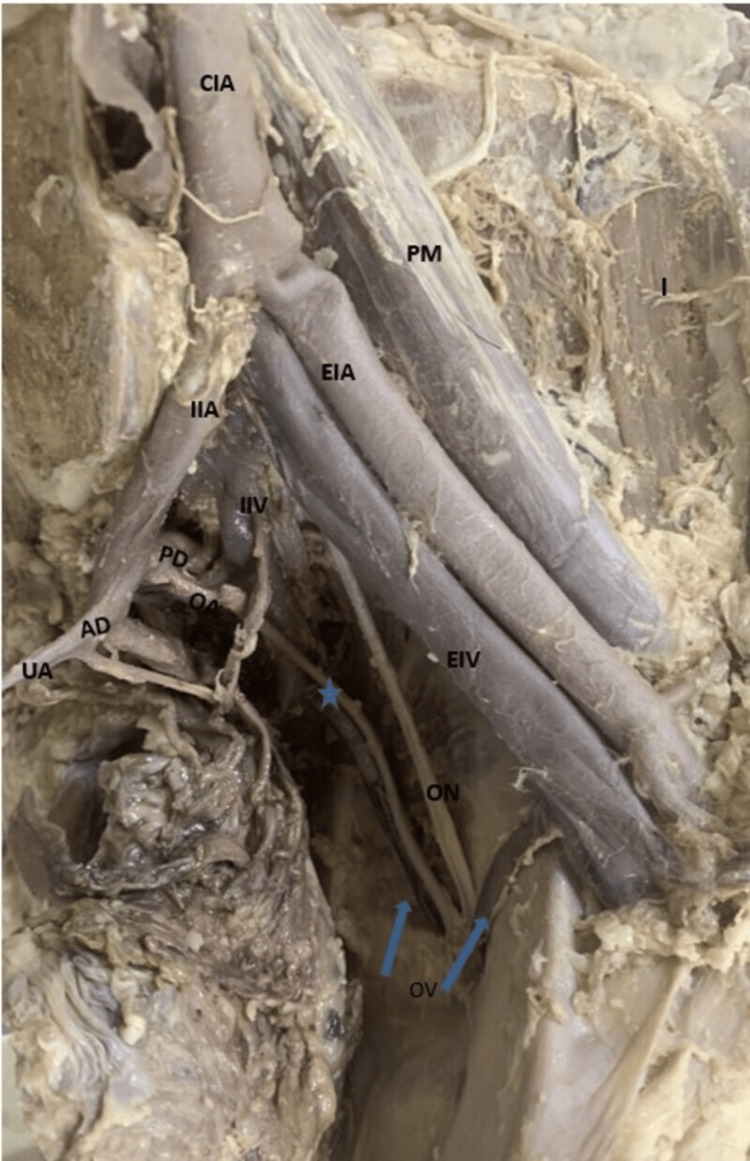
Left side of the pelvis showing the origin of the OA from the PDIIA AD, anterior division; CIA, common iliac artery; EIA, external iliac artery; EIV, external iliac vein; I, iliacus; IIA, internal iliac artery; IIV, internal iliac vein; OA, obturator artery; ON, obturator nerve; OV, obturator vein; PDIIA, posterior division of IIA; PM, psoas major muscle, UA, umbilical artery

In 79.2% (38) of specimens, the OA emerged from the IIA. Among these, 65.8% (25) of specimens showed the OA as a branch of the ADIIA (Figure 1), with the higher prevalence observed in male cadavers (36.8%) (14). In the remaining 12 (31.6%) hemipelvis specimens, the OA was derived as a branch from the posterior division of the IIA (PDIIA), and in one specimen, it originated directly from the main trunk of IIA (Table 2). In 18.8% of the specimens, the OA branched from the EIA either independently or in conjunction with the IEA (Figure 2, Figure 3). Additionally, in 2.6%, the OA originated with the iliolumbar (Figure 4) of the PDIIA. Notably, in one specimen (2%), the OA had dual origins from the IIA and the EIA. When originating from EIA, the OA typically coursed along the posterior surface of the superior pubic ramus in downward, forward, and medial directions. It was lying anterior to the external iliac vein (EIV). After crossing the pelvic brim, OA moved forward and descended into the obturator canal. The ON and obturator vein (OV) entered the canal below the OA. Subsequently, after passing through the obturator canal, it subsequently supplied the adductor compartment of the thigh. However, in one hemipelvis, the OA, originating from the EIA, took a posterior course relative to the EIV before following the described path. Furthermore, we also noticed that across most specimens, venous structures traversed the superior pubic ramus, varying in size and number, extending from the OF to the inguinal region, ultimately draining into the EIV.

**Table 2:** Different types of origin courses of OA from IIA and EIA observed in the present study EIA, external iliac artery; EIV, external iliac vein; IEA, inferior epigastric artery; IIA, internal iliac artery; OA, obturator artery; ON, obturator nerve; OV, obturator vein; SGA, superior gluteal artery

Sr. no	Primary source of origin of OA	Prevalence, N (%)	Secondary source of origin		Male, N (%)	Female, N (%)	OA course		
							Within the lateral wall of the pelvic cavity	Within the obturator canal	Outside of the pelvic cavity
1	IIA	38 (79.2%)	Common trunk		1 (2.6%)	0 (0%)	Length ranges from 38 to 86 mm; passes coursed antero-inferiorly on the lateral pelvic wall with ON above and vein below toward the OF (Figure 1)	One OV and the nerve-related above the artery and the second OV related below the OA in cases of two OVs	Supplies the adductor compartment of the thigh
			Anterior division		14 (36.8%)	11 (29%)	In 90% of specimens, the superior pubic ramus was crossed by a varied number and size of venous structures that coursed from the OF to the inguinal region to drain into the EIV	OV lies below, and the nerve-related above the artery	Supplies the adductor compartment of the thigh
			Posterior division	As separate branch	2 (5.3%)	1 (2.6%)	A long course of OA is seen, lateral to branches of IIA. In one such case, two OVs were seen draining into an internal iliac vein and another into the EIV	OV lies below and the nerve-related above the artery	Supplies the adductor compartment of the thigh
				With SGA	5 (13.2%)	3 (7.9%)			
				With iliolumbar artery	1 (2.6%)	-			
2	EIA	9 (18.8%)	As a separate branch		2 (22.3%)	1 (11.2%)	Length ranges from 28 to 42 mm OA passed anteriorly over the superior ramus of the pubis and turned inwards, passed close to the posterior surface of the superior pubic ramus in downwards, forwards, and medial directions to enter into the obturator canal (Figure 2) medial to femoral ring (N = 2; 22.2%), lateral to femoral ring (N = 7; 77.8%)	ON and the vein entered the canal below the artery	Supplies the adductor compartment of the thigh
			Common origin with IEA		4 (44.2%)	2 (22.3%)	Arose at a distance of 10 mm to 24 mm from the origin point of IEA		
3	Dual origin	1 (2%)	From ADIIA and IEA both		1 (6.7%)	0 (0%)	OA1 arose from ADIIA, while OA2 arose from IEA at a distance of 16 mm from the origin point of IEA, lying lateral to femoral ring	The ON lies above and vein below the OA1, while the ON and the vein enter the canal below OA2	OA1 and OA 2 terminated by supplying in supplied the adductor compartment of the thigh

## Discussion

The IIAs are important in supplying blood to various anatomical structures within the pelvic walls, perineum, external genitalia, gluteal region, and the muscles of the medial compartment of the thigh [16]. The connections between the deep FA and branches originating from the IIAs may develop into distal collateral circulation. Therefore, a comprehensive grasp of the pelvic region vascular anatomy is imperative in surgical procedures conducted at this anatomical site. Anatomists and surgeons have long been intrigued by the diverse variations at the origin of the OA and its course that the literature contains [17]. These variations have significant implications for pelvis procedures, highlighting the importance of detailed anatomical knowledge in ensuring safe and effective surgical outcomes. The most commonly described origin of the OA is typically as a single branch arising from the ADIIA.

Furthermore, the longer course of the OA, resulting from its origin in the PDIIA, may offer additional advantages during grafting procedures. Careful tracing along the aberrant vessel allows surgeons to accurately locate the OF, a pivotal anatomical landmark that ensures adequate inferior dissection of the preperitoneal space. As surgeons employ diverse approaches to the space of Bogros and utilize synthetic mesh anchoring techniques in herniorrhaphies, precise mapping of these vessels becomes increasingly crucial [18].

Our study found that the most frequent origin of OA in both males and females was from the IIA (Table 1), with a higher prevalence from the ADIIA (Table 2). In some cases, it originated from the PDIIA as a separate branch or shared a common origin with the superior gluteal or iliolumbar artery. Bergman et al. noted that the OA originates as a branch of the ADIIA in 41.4% of cases. In 10% of cases, it arises from the superior gluteal artery; in 10%, the interior gluteal artery or internal pudendal trunk; in 4.7%, the inferior gluteal; and in 3.8%, the internal pudendal and in 1.1% of cases from EIA, respectively [19]. Furthermore, we observed one case where the OA arose directly from the main trunk of the IIA. In the study by Biswas et al., which examined 56 pelvic halves, they documented the OA originating from the IEA in 23.2% of cases, significantly less than ours. However, they did not find instances where the OA originated from the IGA, IPA, their standard stem, or the iliolumbar artery [20]. Braithwaite examined 167 specimens and found that the OA originated from the ADIIA in 41% of cases, the most frequent origin observed. Interestingly, he did not encounter a single instance where the OA originated from the PDIIA. The presence of a dual origin for the OA, a rarer anomaly, was reported to occur at an approximate frequency of 2.2%. However, in our present study, dual origins were observed in 6.7% of cases, indicating a slightly higher incidence [21].

The parietal branches of the OA play a significant role as collateral vessels in diseases affecting the aortoiliac and femoral arteries. In instances of ischemic necrosis of the femoral head due to reduced OA blood flow, consideration of a bypass graft connecting the PDIIA to the distal end of the obstruction may be warranted [22]. This will restore adequate blood supply to the affected area and prevent further ischemic damage. In our study, no OA was observed to arise from the CIA. However, the OA originated from the EIA in 18.8% of the specimens studied. Specifically, the study documented that 22.3% of males and 11.2% of females had the OA directly originating from the EIA. This finding contrasts with previous reports. Bergman et al. also noted a relatively frequent variation where the IEA and OA share a common origin, occurring in 20-30% of cases.

In contrast, our study observed a higher incidence of this variation, with 44.2% males and 22.3% females. When the IIA and its branches are ligated during pelvic surgery in women or when there is obstruction of the IIA due to any etiology, the OA and its branches, particularly those supplying the femoral head, remain unaffected if the OA originates from the EIA. This anatomical variation provides a critical safeguard for maintaining essential blood supply in such scenarios.

Embryologically, anomalies in arterial patterns support the fact that during development, there is a selective enlargement or regression process among channels in a primary capillary plexus. The most suitable channels enlarge while others regress and disappear, shaping the final arterial pattern. The OA emerges relatively late during development to supply a plexus that connects with the axial artery of the lower limb alongside the sciatic nerve. The origin of the OA from the PDIIA is attributed to the existence of arterial channels associated with this division, potentially giving rise to the OA. Conversely, arterial channels initially destined for the OA from the ADIIA may regress and disappear. This suggests that before the OA attains independent status as a vessel from the “rete pelvicum,” the blood flow destined for this region follows an unconventional path through its source channels. In contrast to its usual origin from the IIA, the OA can also originate from the IEA or as a direct branch from the EIA. The dual origin of the OA suggests two distinct channels supplying blood flow, one from the IIA and another from the IEA [23].

The “corona mortis” refers to an anatomical variant characterized by the network of a vascular anastomosis connecting the OA and the EIA, IEA, or veins, typically situated on or behind the superior pubic ramus. This anatomical feature is notable because accidental injury to the corona mortis can lead to bleeding, and achieving hemostasis in such cases can be challenging [24]. Therefore, orthopedic surgeons undertaking anterior approaches to the acetabulum, such as the ilioinguinal or intrapelvic approach (modified Stoppa), should proceed cautiously during dissection near the superior pubic ramus. Darmanis et al. also emphasize that while these large retropubic vessels are present in the surgical field, surgeons should proceed cautiously without altering their planned approach solely due to concerns about potential bleeding complications [25]. This ensures safe and effective surgical procedures while minimizing the risk of complications related to vascular anatomy.

## Conclusions

This study found that 48% of cases demonstrated an aberrant origin of the OA, with a higher prevalence in males than females. These anatomical variations, which are well documented in the literature, can have a significant impact on pelvic surgical procedures. Recognizing and assessing these variations during preoperative planning is essential for enhancing surgical outcomes, reducing the risk of iatrogenic complications, and ensuring safer, more effective patient care. Additionally, a thorough understanding of obturator vasculature anatomy is crucial when performing superior gluteal muscle grafts in female patients undergoing breast augmentation. Proper documentation of these variations is vital for aiding surgeons in evaluating and selecting the most appropriate surgical approaches.
